# Association between serum iron and liver transaminases based on a large adult women population

**DOI:** 10.1186/s41043-023-00420-3

**Published:** 2023-07-24

**Authors:** Andong He, Zhuoping Zhou, Lili Huang, Ka Cheuk Yip, Jing Chen, Ruiling Yan, Ruiman Li

**Affiliations:** 1 Department of Obstetrics and Gynecology, The First Affiliated Hospital of Jinan University, No. 613, Huangpu Road West, Tianhe District, Guangzhou, 510630 Guangdong China; 2 Department of Obstetrics and Gynecology, The Sixth Affiliated Hospital of Jinan University, Dongguan Eastern Central Hospital, Dongguan, 523576 Guangdong China; 3 Department of Laboratory Medicine, The First Affiliated Hospital of Jinan University, Guangzhou, Guangdong China; 4 Department of Fetal Medicine, The First Affiliated Hospital of Jinan University, No. 613 Huangpu Road West, Tianhe District, Guangzhou, 510630 Guangdong China

**Keywords:** Iron, Liver transaminases, Women, Nutrition Surveys

## Abstract

**Background:**

Studies are being focused on the potential roles of iron in various diseases, but remain unclear for the association between serum iron and liver injury, especially in adult women.

**Methods:**

Based on the National Health and Nutrition Examination Survey, we investigated the relationship between serum iron and alanine aminotransferase (ALT) and aspartate aminotransferase (AST) among 19,185 adult women.

**Results:**

Using weighted multivariate regression analyses, subgroup analyses, and threshold effect analyses, we found that serum iron was independently and positively correlated with ALT and AST. These associations differed in various age or race. Additionally, we found turning points in the curves of the relationship between serum iron and ALT in all women and the non-pregnant women. Using sensitivity analyses, we further found that the associations between serum iron and the liver transaminases remained positive in the non-pregnant women after adjusting for various covariates, but not in pregnant women. Besides, the positive associations between them kept present after excluding the women with high blood pressure, diabetes, and chronic kidney disease.

**Conclusion:**

The present study indicated a positive association between serum iron and liver transaminases, indicating that serum iron may be a potential biomarker of liver function.

**Supplementary Information:**

The online version contains supplementary material available at 10.1186/s41043-023-00420-3.

## Background

Liver is the largest substantive organ in our body and plays a vital role in the detoxification of xenobiotics as well as the metabolism of nutrients [1]. Various factors, such as alcohol, drugs, and viruses, can cause the liver injury that seriously endangers our health. Hence, the early diagnosis of liver injury is requisite to adopt timely treatment for the maintenance of normal physiological functions [2]. At present, the activity of serum liver transaminases, mainly including alanine aminotransferase (ALT) and aspartate aminotransferase (AST), are used to evaluate the liver injury. However, conventional methods are still barely satisfactory for its diagnosis, so it is necessary to identify more biomarkers for liver injury to combine multiple indicators to improve the reliability of diagnosis.

Iron is one of the essential trace elements for the human body, but excess iron can be harmful, which has been reported to be associated with various human diseases, such as cancers [3], diabetes [4], and alcoholic liver disease [5]. According to a previous study, administration of iron can cause reduction of liver blood flow and acceleration of fibrosis in rats with liver dysfunction [6]. Additionally, it is reported that iron supplementation may increase the levels of ALT and AST in pregnant women [7]. Thus, we speculate that iron levels may be associated with liver function, but with controversial findings having been reported in limited evidence [8]. The relationship between iron and liver function in women also remains unclear. Accordingly, our aim in the present study was to investigate the association between serum iron and liver transaminases based on a representative sample of adult women from the National Health and Nutrition Examination Survey (NHANES).

## Materials and methods

### Study population

**Fig. 1:**
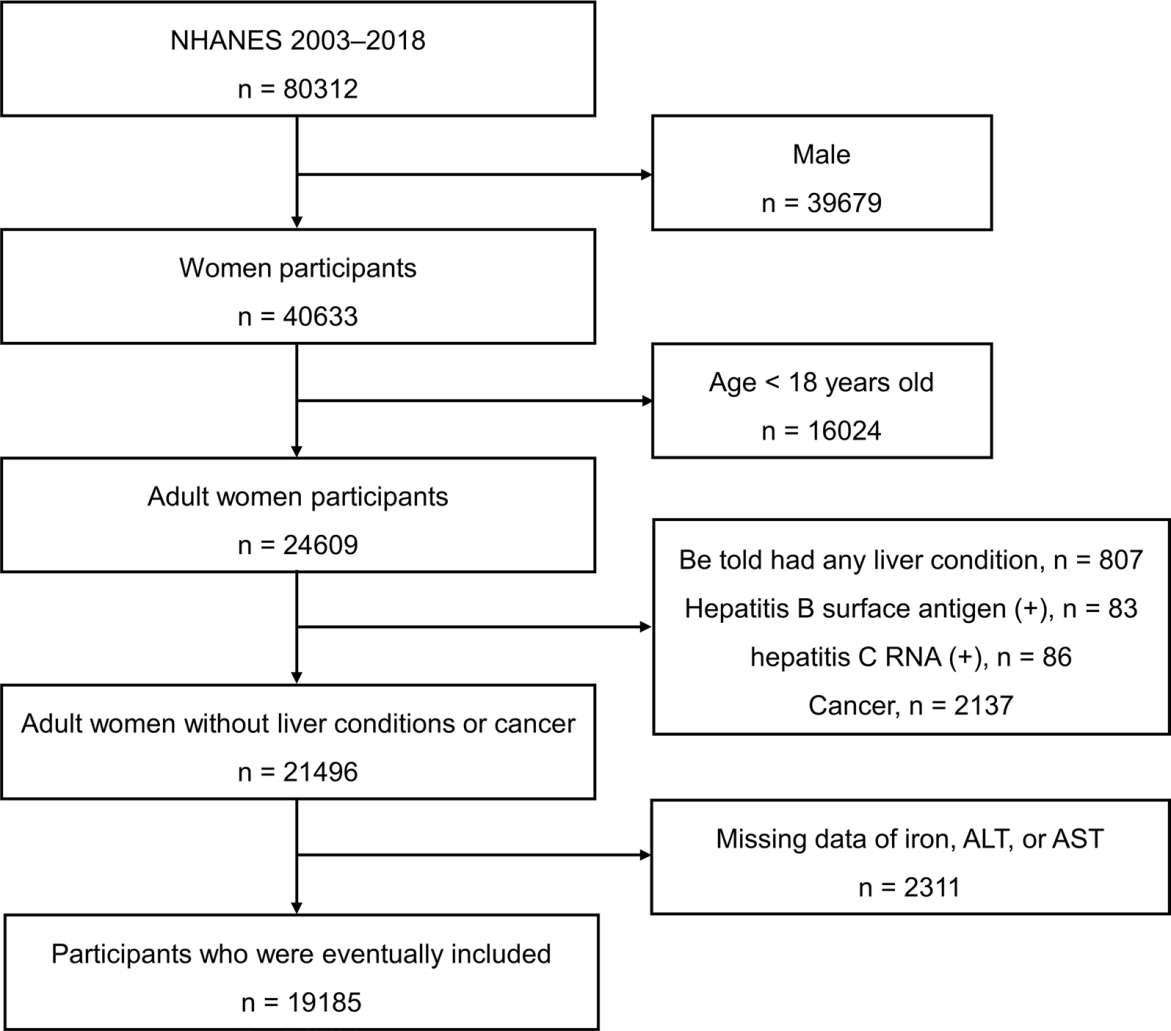
Workflow of the present study. *ALT* alanine aminotransferase, *AST* aspartate aminotransferase, *NHANES* National Health and Nutrition Examination Survey

The data of this study are from the NHANES, a large-scale and nationally representative health survey of the general US population. Details of this database have been described elsewhere [9]. Notably, the NHANES has been approved by the National Center for Health Statistics Ethics Review Board, and all participants gave written informed consent. Data from 2003–2018 of the NHANES were used, and all women participants were included. The excluded criteria were: (1) age < 18 years old; (2) be told had any liver condition, hepatitis B surface antigen (+), hepatitis C RNA (+), or cancer; (3) missing data of iron, ALT, or AST. Eventually, a total of 19,185 participants were included in this cross-sectional study. The process of population screening is shown in Fig. 1.

### Assessments of serum iron and liver transaminases

According to the NHANES, a timed-endpoint method was used to measure serum iron level. Iron is released from transferrin by acetic acid and is reduced to the ferrous state. Then the ferrous ion is immediately complexed with the FerroZine Iron Reagent. The system monitors the changes in absorbance at 560 nm at a fixed-time interval and these changes in absorbance are proportional to the iron concentration in the samples. The Beckman Synchron LX20 or Beckman UniCel® DxC800 Synchron was used in 2003–2016, and the Roche Cobas 6000 (c501 module) analyzer was used in 2017–2018. Additionally, an enzymatic rate method or a kinetic rate method was used to measure the levels of serum ALT and AST using the Beckman Synchron LX20 or the Beckman UniCel® DxC800 Synchron in 2003–2018.

### Covariates

Several continuous variables were included as covariates: age, body mass index (BMI), cholesterol, glucose, and triglycerides. Other covariates were categorical variables, including race (Mexican American, other Hispanic, non-Hispanic white, non-Hispanic black, and other races), education (less than high school, high school or equivalent, more than high school, and unknown), family income-poverty ratio (< 1, ≥ 1 and < 3, ≥ 3, and unknown) [10], pregnancy (yes, no, and unknown), alcohol drinking (moderate drinker, < 1 drink/day; heavy drinker, ≥ 1 drink/day; and unknown) [10], smoking exposure (unexposed, serum cotinine was below the detection limit 0.015 ng/mL; low-exposure, serum cotinine was between the detection limit and 10 ng/mL; and high-exposure, serum cotinine was above 10 ng/mL) [11], work activity (never, moderate, vigorous, and unknown), high blood pressure (HBP), diabetes, and chronic kidney disease (CKD). Among them, HBP was defined as positive responses to the questions “were you told on 2 or more different visits that you had HBP?” and/or “are you now taking prescribed medicine for HBP?”. Diabetes was defined as positive responses to the questions “have you ever been told by a doctor or health professional that you have diabetes?” and/or “are you now taking insulin?” and/or “are you now taking diabetic pills to lower your blood sugar?”. Additionally, CKD was defined as urine albumin/creatinine ratio ≥ 300 mg/g and/or an estimated glomerular filtration rate < 60 mL/1.7 m^2^/min [12, 13]. The details of these covariates are available at http://www.cdc.gov/nchs/nhanes/.

### Statistical analysis

NHANES sample weights were used for calculating all estimates. Weighted multivariate linear regression models were used to investigate the association between serum iron and liver transaminases, and various covariates were adjusted in various models. Weighted linear regression models or weighted chi-square tests was used to evaluate the differences of different groups. Several sensitivity analyses were performed to explore the association between serum iron and liver transaminases in other conditions. The data analyses were performed using packages R (http://www.R-project.org) and EmpowerStats software (http://www.empowerstats.com). A *P* < 0.05 was considered statistically significant.

## Results

### Characteristics of participants

A total of 19,185 adult women were included and their characteristics are shown in Table 1, and the raw clinical data is shown in Additional file 1. These characteristics were subclassified based on serum iron quartiles: the first quartile was 53.00 ug/dL, the second quartile was 73.00 ug/dL, and the third quartile was 96.00 ug/dL. There were significant differences in the characteristics, including age, BMI, race, education, family income-poverty ratio, pregnancy status, alcohol drinking, smoking exposure, work activity, ALT, AST, cholesterol, glucose, triglycerides, HBP, diabetes, and CKD, between the different serum iron quartiles.

**Table 1:** Characteristics of the participants

Iron (ug/dL)	All (n = 19,185)	Q1 (n = 4738)	Q2 (n = 4843)	Q3 (n = 4797)	Q4 (n = 4807)	*P* value
Age (years)	45.71 ± 18.63	42.09 ± 16.10	46.83 ± 17.33	47.08 ± 17.39	44.22 ± 17.09	< 0.001
BMI (kg/m^2^)	29.31 ± 7.63	31.18 ± 8.60	30.14 ± 7.71	28.51 ± 6.90	26.45 ± 6.08	< 0.001
Race (%)						< 0.001
Mexican American	17.98	11.58	8.01	7.63	7.80	
Other Hispanic	9.62	6.91	6.03	5.46	4.83	
Non-Hispanic White	39.37	55.64	63.66	68.33	71.84	
Non-Hispanic Black	22.35	19.00	14.60	11.08	7.00	
Other Races	10.68	6.87	7.69	7.50	8.53	
Education (%)						< 0.001
Less than high school	22.89	18.25	16.21	14.38	12.04	
High school or equivalent	20.67	22.97	22.42	21.32	20.61	
More than high school	49.68	54.61	58.50	61.43	63.29	
Unknown	6.77	4.18	2.87	2.86	4.06	
Family income-poverty ratio (%)						< 0.001
< 1	22.58	19.41	15.49	13.92	13.03	
≥ 1 and < 3	37.82	38.23	33.58	35.06	30.31	
≥ 3	31.14	35.81	44.35	43.70	49.96	
Unknown	8.45	6.54	6.58	7.32	6.70	
Pregnancy (%)						< 0.001
Yes	4.50	3.00	2.33	2.53	2.85	
No	45.27	59.05	47.30	47.14	50.79	
Unknown	50.23	37.95	50.38	50.32	46.36	
Alcohol drinking (%)						< 0.001
Moderate drinker	22.90	24.34	27.26	27.12	27.55	
Heavy drinker	30.40	33.54	32.60	34.17	42.27	
Unknown	46.70	42.13	40.14	38.71	30.18	
Smoking exposure (%)						< 0.001
Unexposed	29.18	27.73	31.20	32.30	34.48	
Low-exposure	51.96	52.37	51.28	47.33	44.16	
High-exposure	18.86	19.90	17.52	20.37	21.36	
Work activity (%)						0.013
Never	50.05	47.94	47.09	47.26	45.65	
Moderate	17.18	18.57	18.90	19.89	20.74	
Vigorous	9.10	10.39	9.83	9.11	10.83	
Unknown	23.67	23.10	24.17	23.74	22.77	
ALT (U/L)	20.59 ± 15.81	19.38 ± 11.60	20.80 ± 13.93	21.04 ± 13.85	20.97 ± 15.36	< 0.001
AST (U/L)	22.89 ± 14.85	21.84 ± 10.22	22.76 ± 12.70	22.94 ± 12.63	23.53 ± 15.39	< 0.001
Cholesterol (mg/dL)	195.74 ± 42.07	189.41 ± 39.59	196.47 ± 41.75	199.78 ± 41.52	200.57 ± 41.73	< 0.001
Glucose (mg/dL)	98.68 ± 36.30	97.90 ± 34.11	98.81 ± 35.62	95.85 ± 27.52	92.42 ± 24.49	< 0.001
Triglycerides (mg/dL)	132.75 ± 102.40	130.38 ± 89.34	137.49 ± 130.08	132.80 ± 92.86	122.47 ± 92.71	< 0.001
HBP (%)	28.38	25.10	29.32	26.57	20.99	< 0.001
Diabetes (%)	10.86	10.29	10.47	7.70	4.89	< 0.001
CKD (%)	2.32	2.24	1.91	1.08	1.06	< 0.001

Mean ± SD for continuous variables: *P* value was calculated by weighted linear regression model; (%) for Categorical variables: *P* value was calculated by weighted chi-square test

*ALT* alanine aminotransferase, *AST* aspartate aminotransferase, *BMI* body mass index, *HBP* high blood pressure, *CKD* chronic kidney disease

### Associations between serum iron and liver transaminases

Using a multivariate regression analysis, we found that serum iron was positively associated with ALT in the model 1 (*β* = 0.015, 95%CI: 0.009–0.020, *P* < 0.001), the model 2 (*β* = 0.026, 95%CI: 0.020–0.032, *P* < 0.001), and the model 3 (*β* = 0.026, 95%CI: 0.020–0.031, *P* < 0.001). This positive correlation persisted after converting serum iron concentrations to quartiles (*P* < 0.001) and the *P* for trend for the three models were also lower than 0.001. Participants with the highest quartile of serum iron had 1.598, 2.575, and 2.540 U/L higher ALT than those with the lowest quartile in the three models, respectively. In the subgroup analysis, the association between serum iron and ALT remained positive in women who are 35–44 years old in the model 2 and model 3, and 45–54 and ≥ 55 years old in all models. This association remained positive across almost all races when stratified by race (Table 2).

**Table 2:** The association between serum iron and ALT

	Model 1, β (95% CI)	Model 2, β (95% CI)	Model 3, β (95% CI)
Iron (ug/dL)	0.015 (0.009, 0.020)***	0.026 (0.020, 0.032)***	0.026 (0.020, 0.031)***
Quartiles of iron			
Q1 (lowest quartile)	Reference	Reference	Reference
Q2	1.428 (0.854, 2.003)***	1.435 (0.869, 2.002)***	1.415 (0.849, 1.981)***
Q3	1.667 (1.099, 2.235)***	2.095 (1.529, 2.661)***	2.051 (1.485, 2.617)***
Q4	1.598 (1.040, 2.155)***	2.575 (2.007, 3.143)***	2.540 (1.972, 3.108)***
* P* for trend	< 0.001***	< 0.001***	< 0.001***
Stratified by age (years) #			
18–24	− 0.003 (− 0.016, 0.010)	0.013 (− 0.000, 0.026)	0.013 (− 0.000, 0.026)
25–34	0.001 (− 0.015, 0.016)	0.014 (− 0.002, 0.030)	0.015 (− 0.002, 0.031)
35–44	0.005 (− 0.008, 0.018)	0.019 (0.006, 0.032)**	0.019 (0.006, 0.032)**
45–54	0.049 (0.034, 0.063)***	0.062 (0.047, 0.076)***	0.061 (0.047, 0.076)***
≥ 55	0.026 (0.018, 0.035)***	0.032 (0.024, 0.041)***	0.032 (0.023, 0.040)***
Stratified by race #			
Mexican American	0.005 (− 0.017, 0.028)	0.027 (0.004, 0.050)*	0.027 (0.005, 0.050)*
Other Hispanic	0.035 (0.010, 0.060)**	0.045 (0.020, 0.070)***	0.044 (0.019, 0.069)***
Non-Hispanic White	0.009 (0.002, 0.017)*	0.023 (0.015, 0.030)***	0.022 (0.014, 0.030)***
Non-Hispanic Black	0.029 (0.019, 0.039)***	0.030 (0.019, 0.040)***	0.029 (0.019, 0.040)***
Other Races	0.024 (0.012, 0.037)***	0.035 (0.022, 0.048)***	0.034 (0.021, 0.046)***

Bold indicates a statistical difference. *P < 0.05, **P < 0.01, ***P < 0.001. Model 1 was not adjusted. Model 2 was adjusted for age, race, education, family income-poverty ratio, pregnancy status, BMI, alcohol drinking, smoking exposure, cholesterol, glucose, triglycerides, and work activity. Model 3 was adjusted for model 2 plus histories of HBP, diabetes, and CKD. # In the subgroup analyses stratified by age and race, the models were not adjusted for the stratification variables themselves

*ALT* alanine aminotransferase, *BMI* body mass index, *HBP* high blood pressure, *CKD* chronic kidney disease

Additionally, serum iron was positively correlated to AST in all models (model 1: *β* = 0.019, 95%CI: 0.013–0.024, *P* < 0.001; model 2 and model 3: *β* = 0.019, 95%CI: 0.014–0.025, *P* < 0.001) (Table 3). The quartiles of serum iron were also positively correlated to AST in all models and the *P* for trend were lower than 0.001. Participants with the highest quartile of serum iron had 1.693, 1.568, and 1.548 U/L higher AST than those with the lowest quartile in the three models, respectively. A positive association between serum iron and AST was found in women who are 45–54 and ≥ 55 years old. This association remain positive mainly in the non-Hispanic White, non-Hispanic Black, other Hispanic, and other races when stratified by race.

**Table 3:** The association between serum iron and AST

	Model 1, β (95% CI)	Model 2, β (95% CI)	Model 3, β (95% CI)
Iron (ug/dL)	0.019 (0.013, 0.024) ***	0.019 (0.014, 0.025) ***	0.019 (0.014, 0.025) ***
Quartiles of iron			
Q1 (lowest quartile)	Reference	Reference	Reference
Q2	0.922 (0.383, 1.462)***	0.552 (0.013, 1.091)*	0.540 (0.001, 1.079)*
Q3	1.106 (0.573, 1.640)***	0.782 (0.244, 1.321)**	0.761 (0.222, 1.300)**
Q4	1.693 (1.170, 2.216)***	1.568 (1.028, 2.109)***	1.548 (1.008, 2.089)***
* P* for trend	< 0.001***	< 0.001***	< 0.001***
Stratified by age (years) #			
18–24	0.002 (− 0.006, 0.011)	0.005 (− 0.003, 0.014)	0.005 (− 0.003, 0.014)
25–34	0.011 (− 0.004, 0.027)	0.013 (− 0.003, 0.029)	0.014 (− 0.003, 0.030)
35–44	0.006 (− 0.006, 0.018)	0.009 (− 0.003, 0.021)	0.009 (− 0.003, 0.021)
45–54	0.053 (0.037, 0.070)***	0.052 (0.036, 0.069)***	0.052 (0.035, 0.069)***
≥ 55	0.022 (0.015, 0.029)***	0.023 (0.016, 0.030)***	0.023 (0.016, 0.030)***
Stratified by race #			
Mexican American	0.014 (− 0.008, 0.036)	0.022 (− 0.001, 0.045)	0.023 (0.000, 0.045)
Other Hispanic	0.027 (− 0.001, 0.055)	0.034 (0.006, 0.063)*	0.035 (0.006, 0.064)*
Non-Hispanic White	0.016 (0.009, 0.023)***	0.017 (0.009, 0.024)***	0.016 (0.009, 0.024)***
Non-Hispanic Black	0.030 (0.020, 0.039)***	0.024 (0.015, 0.034)***	0.024 (0.015, 0.034)***
Other Races	0.016 (0.006, 0.026)**	0.020 (0.010, 0.030)***	0.019 (0.009, 0.029)***

Bold indicates a statistical difference. *P < 0.05, **P < 0.01, ***P < 0.001. Model 1 was not adjusted. Model 2 was adjusted for age, race, education, family income-poverty ratio, pregnancy status, BMI, alcohol drinking, smoking exposure, cholesterol, glucose, triglycerides, and work activity. Model 3 was adjusted for model 2 plus histories of HBP, diabetes, and CKD

^#^In the subgroup analyses stratified by age and race, the models were not adjusted for the stratification variables themselves

*AST* aspartate aminotransferase, *BMI* body mass index, *HBP* high blood pressure, *CKD* chronic kidney disease

**Fig. 2:**
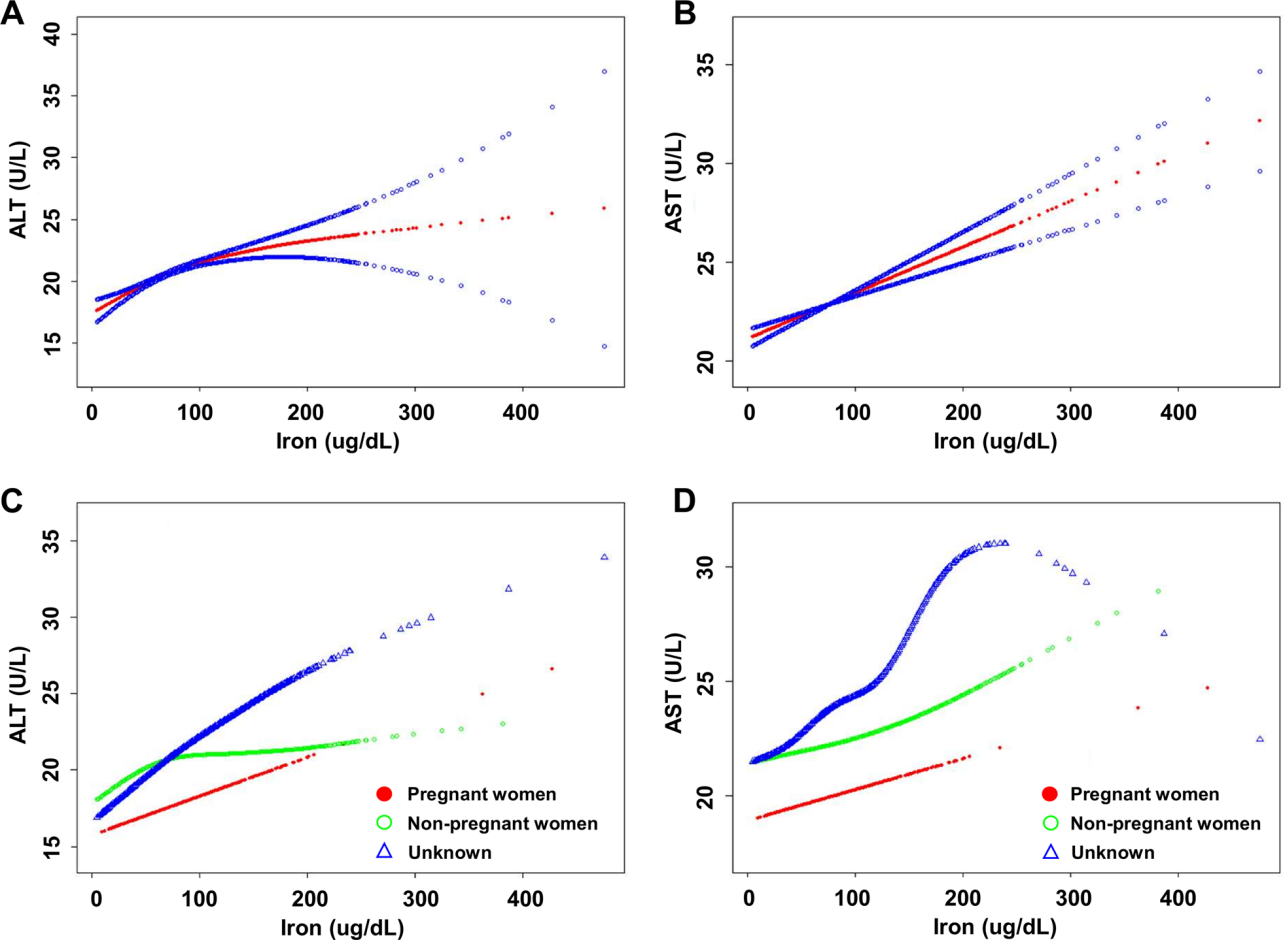
Associations between serum iron and liver transaminases in all participants and stratified by pregnancy status. **A**, **B** Associations between serum iron and ALT and AST in all participants. Age, race, education, family income-poverty ratio, pregnancy status, BMI, alcohol drinking, smoking exposure, cholesterol, glucose, triglycerides, work activity, HBP, diabetes, and CKD were adjusted. **C**, **D** Associations between serum iron and ALT and AST in various pregnancy status. Age, race, education, family income-poverty ratio, BMI, alcohol drinking, smoking exposure, cholesterol, glucose, triglycerides, work activity, HBP, diabetes, and CKD were adjusted. *ALT* alanine aminotransferase, *AST* aspartate aminotransferase, *BMI* body mass index, *CKD* chronic kidney disease, *HBP* high blood pressure

Furthermore, we used the smooth curve fittings to characterize the association between serum iron and liver transaminases, which are shown in Fig. 2. There were positive linear relationships between serum iron and ALT or AST, and there was an inflection point in the relationship between serum iron and ALT, after which the curve tended to be relatively flat (Fig. 2A, B). Therefore, we used a two-piecewise linear regression model to identify the point of inflection which was 68.00 ug/dL in the threshold effect analysis of serum iron on ALT in all participants (Table 4). For a serum iron was lower than 68.00 ug/dL, every 1 ug/dL increase in serum iron was associated with a 0.062 U/L higher ALT (95%CI: 0.045–0.078, *P* < 0.001); additionally, a 1 ug/dL increase in serum iron was associated with a 0.013 U/L increase in ALT (95%CI: 0.005–0.021, *P* = 0.001) when serum iron was higher than 68.00 ug/dL. Notably, we also used the smooth curve fittings to investigate the associations between serum iron and liver transaminases when stratified by pregnancy status, and found that serum iron was positively correlated with ALT and AST in both pregnant and non-pregnant women; however, there was an inflection point in the relationship between serum iron and ALT in non-pregnant women, after which the curve tended to flatten (Fig. 2C, D). According to the results of the two-piecewise linear regression model, the point of inflection was 41.00 ug/dL in the threshold effect analysis of serum iron on ALT in non-pregnant women (Table 4). For a serum iron was lower than 41.00 ug/dL, every 1 ug/dL increase in serum iron was associated with a 0.118 U/L higher ALT (95%CI: 0.052–0.184, *P* < 0.001) in non-pregnant women; additionally, a 1 ug/dL increase in serum iron was associated with a 0.009 U/L increase in ALT (95%CI: − 0.001 to 0.019, *P* = 0.063) in non-pregnant women when serum iron was higher than 41.00 ug/dL.

**Table 4:** Threshold effect analysis of serum iron on ALT in all participants and non-pregnant women using two-piecewise linear regression models

	Adjusted *β* (95% CI), *P* value
All participants	
Fitting by the standard linear model	0.026 (0.020, 0.031), < 0.001
Fitting by the two-piecewise linear model	
Inflection point	68.00 ug/dL
Serum iron < 68.00 ug/dL	0.062 (0.045, 0.078), < 0.001
Serum iron > 68.00 ug/dL	0.013 (0.005, 0.021), 0.001
Log likelihood ratio	< 0.001
Non-pregnant women	
Fitting by the standard linear model	0.016 (0.008, 0.025), < 0.001
Fitting by the two-piecewise linear model	
Inflection point	41.00 ug/dL
Serum iron < 41.00 ug/dL	0.118 (0.052, 0.184), < 0.001
Serum iron > 41.00 ug/dL	0.009 (− 0.001, 0.019), 0.063
Log likelihood ratio	0.002

For all participants, the model was adjusted for age, race, education, family income-poverty ratio, pregnancy status, BMI, alcohol drinking, smoking exposure, cholesterol, glucose, triglycerides, work activity, HBP, diabetes, and CKD. For non-pregnant women, the model was adjusted for age, race, education, family income-poverty ratio, BMI, alcohol drinking, smoking exposure, cholesterol, glucose, triglycerides, work activity, HBP, diabetes, and CKD

### Sensitivity analysis

To further validate the associations between serum iron and liver transaminases in different pregnancy status, we performed a sensitivity analysis and the results demonstrated that there was no significant correlation between serum iron and ALT or AST in pregnant women (Table 5A, B). However, the correlations between serum iron and the two liver transaminases remained positive in non-pregnant women in model 2 and model 3, which were adjusted for various covariates (Table 5C, D). Moreover, we also performed a sensitivity analysis by excluding the participants with HBP, diabetes, and CKD, and found that serum iron was still positively correlated with ALT and AST in model 1 and model 2 (Table 5E, F).

**Table 5:** Associations between serum iron and liver transaminases in the sensitivity analyses

	Model 1, β (95% CI)	Model 2, β (95% CI)	Model 3, β (95% CI)
A	0.009 (− 0.009, 0.026)	0.006 (− 0.012, 0.024)	0.007 (− 0.011, 0.025)
B	0.010 (− 0.003, 0.023)	0.004 (− 0.010, 0.018)	0.004 (− 0.010, 0.018)
C	0.001 (− 0.008, 0.009)	0.016 (0.008, 0.025)***	0.016 (0.008, 0.025)***
D	0.007 (− 0.001, 0.015)	0.009 (0.001, 0.017)*	0.009 (0.001, 0.017)*
E	0.010 (0.003, 0.016)***	0.024 (0.017, 0.030)***	**/**
F	0.015 (0.009, 0.020)***	0.015 (0.010, 0.021)***	**/**

Bold indicates a statistical difference. *P < 0.05, **P < 0.01, ***P < 0.001. A: The association between serum iron and ALT in pregnant women. B: The association between serum iron and AST in pregnant women. C: The association between serum iron and ALT in non-pregnant women. D: The association between serum iron and AST in non-pregnant women. E: The association between serum iron and ALT after excluding participants with HBP, diabetes, and CKD. F: The association between serum iron and AST after excluding participants with HBP, diabetes, and CKD. In the A, B, C, and D, model 1 was not adjusted; model 2 was adjusted for age, race, education, family income-poverty ratio, BMI, alcohol drinking, smoking exposure, cholesterol, glucose, triglycerides, and work activity; and model 3 was adjusted for model 2 plus histories of HBP, diabetes, and CKD. In the E and F, model 1 was not adjusted, and model 2 was adjusted for age, race, education, family income-poverty ratio, pregnancy status, BMI, alcohol drinking, smoking exposure, cholesterol, glucose, triglycerides, and work activity

*ALT* alanine aminotransferase, *AST* aspartate aminotransferase, *BMI* body mass index, *HBP* high blood pressure, *CKD* chronic kidney disease

## Discussion

Liver injury is common and harmful for human health worldwide. In addition to ALT and AST, several novel potential markers of liver injury were unearthed recently [14, 15]. In the present study, we investigated the associations between serum iron and ALT and AST among 19,185 US adult women based on the NHANES data. The results showed that serum iron levels were positively correlated with ALT and AST both without adjustment for any covariates and after adjustment for various covariates. Additionally, this positive association was remained in the non-pregnant women.

It is universally acknowledged that alcohol drinking and smoking can be harmful to liver [16], but in fact there are many factors that may affect liver function, such as BMI, glucose, triglycerides, cholesterol, or blood pressure [17, 18]. A study found that liver function deteriorates with age [19]. Various races may have different patterns of changes in liver transaminases [20], and previous studies found a strong association between race-ethnicity with body iron after adjusting for sociodemographic and lifestyle variables [21]. Using NHANES data, Sakharkar et al. [22] found that pre-diabetic and diabetic status were associated with high levels of ALT and AST among adults. Therefore, these potential confounding factors were included in the regression analysis in this study. Although there may be no evidence that several other factors, such as education, are related to liver function, they were still explored in this study. Notably, transient physiological changes in liver function may occur during pregnancy, but elevated liver transaminases may be due to pregnancy-specific disorders, such as preeclampsia and cholestasis of pregnancy, so pregnancy status was also a focus of this study. We observed a positive association between iron and ALT or AST in both pregnant and non-pregnant women (Fig. 2C, D); however, serum iron and ALT or AST remained significant positive association in the non-pregnant women, but not in the pregnant women according to the sensitivity analysis (Table 5), suggesting that there may be a difference in regulation of iron between pregnant and non-pregnant women, and liver transaminases change more sensitively to changes in iron concentration in non-pregnant women. Iron homeostasis is important for normal pregnancy, and insufficient or excess iron may lead to adverse pregnancy outcomes. The concentration of serum iron will gradually decrease in normal pregnancy, but it can increase under the influence of inflammation and other factors [23]. The iron balance during pregnancy is delicately regulated, possibly involving mutual regulation among the mother, placenta, and fetus, but the underlying mechanism is not fully understood [24].

Under normal conditions, serum iron binds to transferrin, which is then delivered to the bone marrow [25, 26]. However, non-transferrin-bound iron will accumulate when the level of serum iron exceeds the buffering capacity of transferrin, and its accumulation in hepatocytes may be toxic for liver [27, 28]. The high redox potential of iron may be the basis of its toxicity since excessive iron could cause accumulation of toxic reactive oxygen species and increased oxidative stress, leading to cell damage and ultimately cell death [29]. Recently, iron-dependent ferroptosis, characterized by a decreased glutathione and increased lipid peroxidation, is getting more attention. Several studies indicated that ferroptosis may be involved in cancers [30, 31], ischemia–reperfusion injury [32], and renal damage [33, 34]. Yu et al. [28] found that patients with liver cirrhosis have lower levels of serum transferrin and hepatic transferrin, and higher levels of hepatic iron and lipid peroxidation. They also found that ferroptosis may play a role in the liver damage of mice. Additional study also found that iron accumulation and ferroptosis play roles in the pathogenesis of acute liver injury in the mice model, and ferrostatin-1, which can inhibit ferroptosis, ameliorated liver dysfunction via reducing iron [35]. Therefore, the positive correlation between serum iron and liver transaminases may be due to the toxicity of elevated iron to hepatocytes, and ferroptosis may be one of the pathways of its pathological mechanism. However, more experiments are required to explore the pathogenesis of elevated serum iron in liver injury.

There are several limitations of the present study, for example, all data were from the NHANES, which is designed to provide nationally representative estimates of US population, so a further investigation for other population is required. Additionally, we could not infer the causality between serum iron and liver transaminases as this is a large cross-sectional study. Although we have adjusted for several potentially influential covariates in our linear regression analysis, we cannot rule out all confounding factors for the study results. It is expected that prospective studies will be conducted to further determine the relationship between serum iron and liver transaminases.

## Conclusion

Serum iron was independently and positively correlated with ALT and AST among US adult women. The associations may be affected by various age or race. Pregnancy status may also affect the results, and the present results showed that the positive association between serum iron and liver transaminases was present among non-pregnant women, but not in pregnant women. Furthermore, the association between them remained positive after excluding participants with HBP, diabetes, and CKD. However, more studies are warranted to elucidate the potential mechanisms underlying these associations between serum iron and liver transaminases.

## Supplementary Information

**Additional file 1**: Raw clinical data of the participants in this study.

## Data Availability

The data used in the present study are publicly available on the NHANES (http://www.cdc.gov/nchs/nhanes/).

## Abbreviations

ALT: Alanine aminotransferase
AST: Aspartate aminotransferase
BMI: Body mass index
CKD: Chronic kidney disease
HBP: High blood pressure
NHANES: National Health and Nutrition Examination Survey
